# “Degrees of Freedom”: Comparing Mental Distress of Populations with Different Levels of Access to Care-Prisoners, Psychiatric Patients and General Population

**DOI:** 10.3390/healthcare10091726

**Published:** 2022-09-08

**Authors:** Maayan Nagar

**Affiliations:** 1 School of Criminology, Faculty of Law, University of Haifa, Haifa 3498838, Israel; mbeerinag@staff.haifa.ac.il; 2 Department of Criminology, Faculty of Humanities and Social Sciences, Ariel University, Ariel 40700, Israel

**Keywords:** psychiatric care, prisoners’ mental health, general health questionnaire, representative sample

## Abstract

Objectives: The study presents an analysis of the risk for common mental disorders (CMDs) in populations with different levels of access to mental health care. Methods: We merged and statistically compared the representative data of prisoners to data collected from psychiatric clinics and the general population. Participants across all samples completed the General Health Questionnaire. Results: More than half of the inmates met the criteria for CMDs, while rates were 25% in the general population and 80% among psychiatric patients. The odds of prisoners being five times more likely to meet the criteria for CMDs were five times higher than the odds of the general population while controlling for demographic variables. Conclusions: The study highlights the need for prisoners for mental health services. Prisoners face stressful life conditions before and during incarceration while having limited access to medical and psychological treatment stresses the need for systemic interventions.

## 1. Introduction

Common mental health disorders (CMDs), mainly depression and anxiety, are a global burden. Studies conducted among prisoners in different countries show high prevalence of CMDs [1,2]. CMDs are highly comorbid with other mental disorders such as schizophrenia, substance use and personality disorders among prisoners [3]. Surprisingly, mental health problems among prisoners usually go undetected and untreated [1]. Access to care is controlled by the prison service, which usually offers very limited treatment programs. It is precisely in these places, where the system decides what the individual is exposed to, that treatment could be provided to individuals that otherwise will not actively seek treatment. Overall, even though incarceration could be an opportunity to treat and reform prisoners, studies generally do not show improvement in mental health among prisoners [4].

The elevated risk for CMDs among prisoners can be attributed to many factors. One relates to the incarceration itself, including the loss of personal freedom, the loss of social support and relationships, and the change of employment, social status, and power [5]. Another factor relates to the physical conditions of prisons involving violence, lack of privacy, and neglect of physical and mental health [4]. Finally, low socioeconomic status, lower education, being unmarried, history of mental health disorders, major life changes, stressors, and substance abuse have been associated with elevated risk for CMDs [6,7].

Poor mental health during the incarceration period and post-release from prison have been linked to a higher likelihood of recidivism [8]. This topic is of particular interest due to The First Step Act (FSA) signed by the Trump administration in December 2018. The law emphasizes the need for the development of risk and needs assessment and rehabilitation programs aimed to improve prisoners’ mental health and reduce recidivism [9]. To date, most research and policy focused primarily on post-release rehabilitation efforts and less attention was paid to the incarceration period.

In the current study, an analysis of CMDs was performed using data from a representative sample of inmates from prisons in Israel. In order to compare between populations with different levels of access to care, inmate (no access to care) data were statistically compared to data collected from psychiatric clinics (access to care) and the general population (access to care). To the best of my knowledge, there are no current studies comparing the representative data of those three populations.

## 2. Method

### 2.1. Sample

*Prisoner sample:* A multi-stage random stratified cluster sampling approach was applied. First, we randomly sampled six prisons from three strata according to their size, security level (prison security levels refer to the physical security parameters of the prison, the staff-to-inmate ratio, and the freedoms afforded to inmates; generally, the higher the security level, the more restrictions are placed on inmates) and representation in the country (three from Central, two from North, and one from South Israel; two maximum and three medium-security prisons). Then, we randomly selected two to four wards (ordinary wards, no special characteristics) from each prison (clusters). All prisoners in the ward completed the survey. The study applied a multi-round cross-sectional design, and data collection was performed in three rounds from 2018–2019 as a part of a larger study. The response rate was 80.5% and resulted in a total of 527 participants.

*Psychiatric patients sample*: A total of 302 consecutive adult psychiatric patients who presented for a first ever or a renewed episode of care in four psychiatric clinics in two large cities in Israel during 2012 (For full description see [10]).

*General population sample*: Data were based on the Israeli component of the WMHS of noninstitutionalized adults collected during 2004. The sample population was extracted from the National Population Register (NPR) and comprised noninstitutionalized de jure residents aged 21 years and older. The sample was designed to reflect the distribution of selected gender-age-ethnicity groups in the general population. The overall response rate was 73% totaling 4859 completed interviews (For full description see [11]).

### 2.2. Participants across All Samples Signed Informed Consent

The authors assert that all procedures contributing to this work comply with the ethical standards of the relevant national and institutional committees on human experimentation and with the Helsinki Declaration of 1975, as revised in 2008. All procedures involving human subjects/patients were approved by the relevant Institutional Ethics Committees (IRB of Bar Ilan University, Department of Criminology, Faculty of Social Sciences, for prisoner data collection; Helsinki committees for the psychiatric patient sample).

## 3. Materials and Procedure

The 12-item General Health Questionnaire (GHQ-12; [12]) is a widely used and validated screening instrument for CMDs. Items are rated on a Likert scale ranging from 0-‘not at all’ to 3-‘much more than usual’. A score of 1 was assigned to ‘rather more’ or ‘much more than usual’ and 0 to ‘not at all’ or ‘no more than usual’ responses. Final score was computed as the sum of all items (range 0 to 12). Scores of four or more were set to indicate GHQ ‘caseness’: a case of CMD that is the most accepted convention, indicating a possibility for a CMD and a need for assessment and treatment [13,14]. Participants responding to less than four items were coded as missing.

## 4. Statistical Analysis

Data collected from prisons were merged with population data and data from psychiatric clinics. This allowed for a direct comparison between the samples. Chi-Square tests were computed to compare categorical variables, while *t*-tests were computed for continuous variables. A binary logistic regression was computed to predict GHQ ‘caseness’ and a linear regression was computed to predict a GHQ sum score between the three samples, while accounting for the potential confounding effects of sociodemographic factors (age, gender, years of education, marital status, and migration). To examine gender differences, *t*-tests and χ2 analyses were computed. Participants with missing data in demographic variables were excluded from the analysis (inmate sample 19.7% *n* = 104, psychiatric patients 10.3% *n* = 31, population 0.1%, *n* = 7).

## 5. Results

The characteristics of the samples are presented in Table 1. More than half of the inmates participating in the study met the criteria for GHQ ‘caseness’, while the rates were approximately 25% in the general population and about 80% from the psychiatric patient sample.

A binary logistic regression analysis predicting GHQ ‘caseness’ showed significant results (χ2 (8) = 654.23, *p* < 0.001, Cox and Snell R2 = 0.11, Nagelkerke R2 = 0.16). The odds to meet the criteria of GHQ ‘caseness’ were five times higher in the inmate group as compared with the odds in the general population (Adjusted OR = 4.88, 95% CI 3.90–6.11), while psychiatric patients were approximately 13 times more likely to meet the criteria of the GHQ ‘caseness’ as compared with the general population (Adjusted OR = 12.98, 95% CI = 9.51–17.72). A linear regression analysis predicting the GHQ total score showed significant results (F (85,532) = 145.13, *p* < 0.001, R2 = 0.17). Inmates scored 1.8 higher than the general population on the GHQ (β = 0.18, t = 13.85, *p* < 0.001), while psychiatric patients scored 4.0 higher than the general population (β = 0.33, t = 26.46, *p* < 0.001). Other significant predictors of higher emotional distress (both GHQ ‘caseness’ and GHQ mean score) included being female, immigrant, older, and being without a romantic partner (single, separated, divorced or widowed). See Table 2 for coefficients.

We further examined gender differences in emotional distress and GHQ ‘caseness’ among each group (inmates, psychiatric patients and general population). Significant gender differences in emotional distress were only found in the general population, with females reporting higher emotional distress (both higher GHQ mean score and GHQ ‘caseness’) than males (see Table 3). No significant gender differences were found among inmates and psychiatric patients.

## 6. Discussion

The current study focused on populations with different levels of access to mental health care, and directly compared a large representative sample of the inmate population to two representative samples, namely the general population sample and the psychiatric patient sample. Approximately half of the prisoners met the criteria for mental distress, as compared with 24% in the general population and 80% among psychiatric patients. The odds of prisoners to meet the criteria for mental distress were five times higher than the odds of the general population, while controlling for demographic variables. Prisoners scored significantly higher than the general population on the GHQ total score, and significantly lower than psychiatric patients. These results place inmates at a high risk for mental health disorders, with very limited access to care compared with the general population and psychiatric patients. The results are consistent with previous studies showing high rates of mental health disorders among inmates [1,2,14]. This study expands findings to CMDs and not only depression, while providing a first comparison of representative data from inmates to the general population and to psychiatric patients, while statistically controlling for confounding variables.

The natural course of mental distress in prisons has only been examined in a few studies. Those studies show that prisoners have high levels of mental health problems, either prior to or due to incarceration. They also show that prisoners with pre-incarceration mental health problems show improvement in symptoms during imprisonment [15,16]. This may be due to the reporting of these psychiatric disorders at the time of admission. However, prisoners that develop mental health symptoms during incarceration often go undetected and untreated. Overall, duration of imprisonment appears to have no significant impact on mental health [4]. Notably, mental health problems and psychiatric disorders (mainly depression, bipolar disorder, and schizophrenia) have been linked to an elevated risk of suicide among prisoners [17,18], highlighting the importance of interventions and therapy for this vulnerable population. There is also a lack of studies about the effects of mental health treatments in prison settings, mainly due to several reasons including problems with obtaining permissions and running interventions, lack of funding, and the division of prisoner health from public health [1]. There is some evidence for the effects of psychological interventions, but reported effect sizes are not large, and it is unclear whether any improvements are sustained [1]. Another way of improving mental health and reducing inmate recidivism is by providing work programs, vocational education, and training. As can be seen in our study, lower education relates to greater emotional distress. Inmates have relatively low years of education as compared with the general population. Many studies have documented the positive effects of education and training of inmates on reducing recidivism rates and improving mental health [19,20,21,22]. Taken together with the results of the current study, the inmate population, which has no access to mental health treatment outside of the prison, cannot get appropriate treatment within the prison and thus may be at great risk for self-harm. Correctional facilities have the sole responsibility for inmates’ mental health. They should provide them with appropriate diagnosis and treatment while offering vocational training and employment options that can also improve the mental health status of inmates

In the current study, we found no significant gender differences in emotional distress among inmates and psychiatric patients, as opposed to the higher emotional distress reported by females (compared to males) in the general population. As for psychiatric patients, it makes sense that people, regardless of gender, seek mental health care when their emotional distress is high. For the inmate sample, it may be that females and males both score high on emotional distress (see, for example [23] regarding depression among prisoners), but their reasons and stressors could be different. For example, women in prison may experience emotional distress due to being separated from their children and families and losing parental roles and social support. In contrast, men may experience stressors related to personal safety, violence, and lack of freedom [24]. Future studies should examine the specific reasons that men and women feel emotional distress in prisons.

Strengths of the current report include a large sample size, a nationally representative sample of the prisoners’ population, and comparison to two other representative samples. In addition, a widely used and well-validated measure for CMDs enabled the comparison of the data.

The study is not without limitations. First, data collected from the general population are dated back to 2004, and data from psychiatric clinics were collected in 2012. The data from 2004 are the only national study conducted in Israel that provides estimation of CMDs in the population; thus, no recent data were available. Even though much advocating for the burden of mental health disorders has been done over the years, studies assessing changes in GHQ-12 scoring over time have shown no significant changes in adult population [25]. No significant health or political-related events took place during the different times of data collection. Second, the inmates’ sample was significantly different from the other samples in demographic variables. We have controlled for the possible confounding effects of relevant variables in our statistical analysis. Even with the sociodemographic differences between the samples, the sample is representative of the inmate population and should be treated as such. Third, since this is a cross-sectional study, there is no way to know whether inmates suffer from CMDs before incarceration or CMDs resulting from the incarceration itself. Studies should assess mental health before, during, and post-incarceration to examine the effects of imprisonment on mental distress. Fourth, we only examined basic sociodemographic factors; future studies should extend our findings and examine additional important variables that contribute to mental distress such as familial risks of psychiatric disorders and related traits (cognitive abilities, personality, etc.). Future studies should also examine the contribution of systemic issues rising from inside (overcrowding, lack of sanitation, etc.) and outside (drug cartels, debts, familial difficulties, etc.) the prison on prisoners’ mental health.

The current study highlights the need for systemic structural change in prisons on the matter of mental health among prisoners. The fact that prisoners face stressful life conditions prior to and during incarceration while having very limited access to medical and psychological treatment stresses the need for systemic awareness and interventions. Exposing fragile populations to imprisonment-related stressors might further marginalize them and deepen their frailty, resulting in possible worse mental status [26]. The study calls for early screening procedures to identify prisoners with CMDs and the development of mental health treatment programs within prison settings.

## Figures and Tables

**Table 1:** Summary and comparisons of demographics and psychological distress among all study samples.

	Inmates Sample (N = 527)	Psychiatric Patients Sample (N = 302)	General Population (N = 4858)	Inmates vs. Psychiatric Patients	Inmates vs. General Population
**Demographics**					
Gender				χ2 (1) = 248.24, *p* < 0.001	χ2 (1) = 301.15, *p* < 0.001
Male	88.9% (468)	36.5% (110)	49% (2379)		
Female	11.2% (59)	63.5% (191)	51% (2479)		
Age	35.0 ± 12.2	40.5 ± 15.5	46.1 ± 17.4	t (523.2) = 5.20, *p* < 0.001	t (779.64) = 18.91, *p* < 0.001
Years of education	10.6 ± 3.2	13.0 ± 3.1	12.8 ± 4.2	t (742) = 10.09, *p* < 0.001	t (738.17) = −14.49, *p* < 0.001
Family status				χ2 (2) = 8.03, *p* = 0.018	χ2 (2) = 316.32, *p* < 0.001
Single	50.3% (238)	40.2% (121)	18.5% (900)		
Married	28.1% (133)	31.9% (96)	66.4% (3228)		
Separated/divorced/widowed	21.6% (102)	27.9% (84)	15.1% (730)		
Migration				χ2 (1) = 0.03, *p* = 0.875	χ2 (1) = 38.79, *p* < 0.001
Yes	29% (153)	29.6% (86)	43.2% (2100)		
No	71% (374)	70.4% (205)	56.8% (2758)		
**Mental distress**					
GHQ ‘caseness’				χ2 (1) = 58.47, *p* < 0.001	χ2 (1) = 195.82, *p* < 0.001
Yes	52.8% (278)	79.5% (240)	24.2% (1174)		
No	47.2% (249)	20.5% (62)	75.8% (3684)		
GHQ mean score	3.8 ± 2.4	6.3 ± 3.1	2.3 ± 2.4	t (511.5) = 12.34, *p* < 0.001	t (5383) = 13.63, *p* < 0.001

**Table 2:** Coefficients and significance levels for logistic and linear regressions predicting emotional distress.

	GHQ ‘Caseness’				GHQ Mean Score		
	B	Sig	OR	95%CI	B	β	Sig
(Constant)	−2.31		0.10		0.82		
Females vs. males	0.43	0.000	1.53	1.35–1.75	0.56	0.11	0.000
Immigrant	0.21	0.005	1.23	1.07–1.42	0.20	0.04	0.008
Years of education	0.00	0.758	1.00	0.99–1.00	0.00	0.00	0.963
Age	0.02	0.000	1.02	1.01–1.02	0.02	0.13	0.000
Single vs. married	0.27	0.004	1.30	1.09–1.56	0.37	0.06	0.000
Divorces/separated/widowed vs. married	0.36	0.000	1.44	1.21–1.71	0.59	0.08	0.000
Inmates vs. General population	1.59	0.000	4.88	3.90–6.11	1.78	0.18	0.000
Psychiatric patients vs. General population	2.56	0.000	12.98	9.51–17.72	4.01	0.33	0.000

**Table 3:** Gender differences in emotional distress among inmates, psychiatric patients and general population.

	Inmates Sample (N = 527)			Psychiatric Patients Sample (N = 302)			General Population (N = 4858)		
	Males (N = 468)	Females (N = 59)	Statistic	Males (N = 110)	Females (N = 191)	Statistic	Males (N = 2377)	Females (N = 2477)	Statistic
GHQ mean score	3.8 ± 2.4	3.7 ± 2.6	t (525) = 0.13	6.6 ± 3.2	6.1 ± 3.0	t (299) = 1.41	1.9 ± 2.3	2.6 ± 2.5	t (4838) = −10.91 ***
GHQ ‘caseness’	53% (248)	50.8% (30)	χ2 (1) = 0.10	78.2% (86)	80.1% (153)	χ2 (1) = 0.16	19.2% (456)	29.9% (718)	χ2 (1) = 63.57 ***

Note. *** *p* < 0.001.

## Data Availability

The data presented in this study are available on request from the corresponding author.
